# Development of a Deep Learning Model to Identify Lymph Node Metastasis on Magnetic Resonance Imaging in Patients With Cervical Cancer

**DOI:** 10.1001/jamanetworkopen.2020.11625

**Published:** 2020-07-24

**Authors:** Qingxia Wu, Shuo Wang, Shuixing Zhang, Meiyun Wang, Yingying Ding, Jin Fang, Qingxia Wu, Wei Qian, Zhenyu Liu, Kai Sun, Yan Jin, He Ma, Jie Tian

**Affiliations:** 1 College of Medicine and Biomedical Information Engineering, Northeastern University, Shenyang, Liaoning, China; 2 CAS Key Laboratory of Molecular Imaging, Institute of Automation, Chinese Academy of Sciences, Beijing, China; 3 Beijing Advanced Innovation Center for Big Data–Based Precision Medicine, School of Medicine and Engineering, Beihang University, Beijing, China; 4 Medical Imaging Center, The First Affiliated Hospital of Jinan University, Guangzhou, China; 5 Department of Medical Imaging, Henan Provincial People's Hospital, Zhengzhou, Henan, China; 6 People's Hospital of Zhengzhou University, Zhengzhou, Henan, China; 7 People's Hospital of Henan University, Zhengzhou, Henan, China; 8 Department of Radiology, the Third Affiliated Hospital of Kunming Medical University, Yunnan Cancer Hospital, Kunming, Yunnan, China; 9 Department of Electrical and Computer Engineering, University of Texas at El Paso; 10 University of Chinese Academy of Sciences, Beijing, China; 11 Engineering Research Center of Molecular and Neuro Imaging of Ministry of Education, School of Life Science and Technology, Xidian University, Xi’an, Shaanxi, China

## Abstract

**Importance:**

Accurate identification of lymph node metastasis preoperatively and noninvasively in patients with cervical cancer can avoid unnecessary surgical intervention and benefit treatment planning.

**Objective:**

To develop a deep learning model using preoperative magnetic resonance imaging for prediction of lymph node metastasis in cervical cancer.

**Design, Setting, and Participants:**

This diagnostic study developed an end-to-end deep learning model to identify lymph node metastasis in cervical cancer using magnetic resonance imaging (MRI). A total of 894 patients with stage IB to IIB cervical cancer who underwent radical hysterectomy and pelvic lymphadenectomy were reviewed. All patients underwent radical hysterectomy and pelvic lymphadenectomy, received pelvic MRI within 2 weeks before the operations, had no concurrent cancers, and received no preoperative treatment. To achieve the optimal model, the diagnostic value of 3 MRI sequences was compared, and the outcomes in the intratumoral and peritumoral regions were explored. To mine tumor information from both image and clinicopathologic levels, a hybrid model was built and its prognostic value was assessed by Kaplan-Meier analysis. The deep learning model and hybrid model were developed on a primary cohort consisting of 338 patients (218 patients from Sun Yat-sen University Cancer Center, Guangzhou, China, between January 2011 and December 2017 and 120 patients from Henan Provincial People's Hospital, Zhengzhou, China, between December 2016 and June 2018). The models then were evaluated on an independent validation cohort consisting of 141 patients from Yunnan Cancer Hospital, Kunming, China, between January 2011 and December 2017.

**Main Outcomes and Measures:**

The primary diagnostic outcome was lymph node metastasis status, with the pathologic characteristics diagnosed by lymphadenectomy. The secondary primary clinical outcome was survival. The primary diagnostic outcome was assessed by receiver operating characteristic (area under the curve [AUC]) analysis; the primary clinical outcome was assessed by Kaplan-Meier survival analysis.

**Results:**

A total of 479 patients (mean [SD] age, 49.1 [9.7] years) fulfilled the eligibility criteria and were enrolled in the primary (n = 338) and validation (n = 141) cohorts. A total of 71 patients (21.0%) in the primary cohort and 32 patients (22.7%) in the validation cohort had lymph node metastais confirmed by lymphadenectomy. Among the 3 image sequences, the deep learning model that used both intratumoral and peritumoral regions on contrast-enhanced T1-weighted imaging showed the best performance (AUC, 0.844; 95% CI, 0.780-0.907). These results were further improved in a hybrid model that combined tumor image information mined by deep learning model and MRI-reported lymph node status (AUC, 0.933; 95% CI, 0.887-0.979). Moreover, the hybrid model was significantly associated with disease-free survival from cervical cancer (hazard ratio, 4.59; 95% CI, 2.04-10.31; *P* < .001).

**Conclusions and Relevance:**

The findings of this study suggest that deep learning can be used as a preoperative noninvasive tool to diagnose lymph node metastasis in cervical cancer.

## Key Points

**Question:**

Can deep learning identify preoperative noninvasive lymph node metastasis diagnosis in cervical cancer?

**Findings:**

This diagnostic study including a total of 479 patients developed a deep learning model to preoperatively and noninvasively identify lymph node metastasis on magnetic resonance imaging, achieving an area under the receiver operating characteristic curve of 0.933 in the independent validation cohort. The predicted lymph node metastasis probability was significantly associated with prognosis of cervical cancer.

**Meaning:**

Findings from this study suggest that deep learning can be used as a preoperative noninvasive tool for diagnosing lymph node metastasis in cervical cancer.

## Teaser

This diagnostic study develops a machine learning tool for use in preoperative noninvasive identification of lymph metastasis on magnetic resonance imaging in patients with cervical cancer.

## Introduction

Cervical cancer is one of the most common cancers among women.^1^ The treatment and management of cervical cancer are often guided by the International Federation of Gynaecology and Obstetrics (FIGO) staging system, which is based on clinical assessment and imaging rather than invasive investigations, such as surgery.^2^ In the 2018 FIGO staging system, once lymph node (LN) metastasis (LNM) is identified either by imaging or pathologic testing, cancer will be considered as stage IIIC irrespective of other findings.^3^ Moreover, LNM has been reported to be associated with prognosis and treatment planning in cervical cancer.^4,5^ Specifically, patients who show evidence of LNM may undergo chemoradiotherapy rather than surgery as their first choice,^6^ avoiding surgery followed by adjuvant chemoradiotherapy and possible serious complications thenceforth.^7,8^ Therefore, accurate identification of LN status preoperatively in patients with cervical cancer might avoid unnecessary surgical intervention and benefit treatment planning.

Magnetic resonance imaging (MRI), a commonly used imaging modality in cervical cancer,^9^ provides a preoperative method for assessing LN status in cervical cancer. However, the traditional methods, which rely mainly on assessing the size of LNs on MRI, have limited sensitivity in diagnosing LNM in cervical cancer and might lead to inappropriate treatment decisions.^10,11,12^ Many attempts have been made to improve the performance of MRI in diagnosing LNM before surgery, for example, using radiomic features that extract the quantitative human-defined image features, such as shape, intensity, and texture features.^13,14,15,16^ In previous research, the sensitivity of MR images to discriminate metastatic from nonmetastatic LN has shown improvement by using radiomic features.^13^ However, radiomic features need time-consuming tumor delineation, and they might not be adaptive to specific clinical issues.

Deep learning (DL) as an artificial intelligence method has recently shown promising performance in many medical image analysis tasks,^17,18,19^ such as diagnosing Alzheimer disease,^20^ screening for breast cancer,^21^ and detecting thoracic diseases.^22^ Moreover, DL also exhibited predictive performance in cervical cancer, such as screening and predicting toxic rectal reactions to radiotherapy.^23,24^ Compared with traditional methods, DL has an advantage in automatically learning and hierarchically organizing task-adaptive image features.^25^ Even though these features cannot be identified visually, they tend to reflect the high-dimensional association between images and clinical issues.^26^ Furthermore, DL does not require precise tumor delineation, making it an easy-to-use method in clinical practice. In many tumor analysis tasks, DL outperforms traditional radiomic features.^27,28,29^ In this research, we aimed to develop a DL model to provide a preoperative noninvasive tool for diagnosing LNM in cervical cancer.

## Methods

Two outcomes were studied. The primary diagnostic outcome was LNM status, with the pathologic characteristics diagnosed by lymphadenectomy. We first developed a DL model that used MR images to diagnose LNM. Then we proposed a hybrid model that integrated tumor image information and MRI-reported LN (MRI-LN) status. Herein, MRI-LN status was defined as positive if the short-axis diameter of the largest LN shown on MRI was equal to or larger than 1 cm.^10^ We assessed the models' performance by receiver operating characteristic analysis. The second primary clinical outcome was disease-free survival (DFS). We assessed the prognostic ability of the hybrid model with regard to DFS by the Kaplan-Meier method.

The institutional review boards of Sun Yat-sen University Cancer Center, Henan Provincial People's Hospital, and Yunnan Cancer Hospital approved this retrospective study with deidentified data, and the need for informed consent from patients was waived. This study followed the Standards for Reporting of Diagnostic Accuracy (STARD) reporting guideline for diagnostic studies.

A total of 479 patients with cervical cancer who underwent radical hysterectomy and pelvic lymphadenectomy were enrolled in this research. A total of 338 patients from Sun Yat-sen University Cancer Center (n = 218, from January 2011 to December 2017) and Henan Provincial People's Hospital (n = 120, from December 2016 to June 2018) composed the primary cohort, and 141 patients from Yunnan Cancer Hospital between January 2011 and December 2017 composed the independent validation cohort. All of these patients met the following inclusion criteria: (1) pathologically confirmed cervical cancer; (2) pelvic MRI performed within 2 weeks before the operation; (3) complete clinicopathologic data available, such as age, FIGO stage, histologic characteristics, differentiation, lymphovascular space invasion, LNM, and MRI-LN status; (4) no concurrent cancers; and (5) no preoperative treatment. We excluded patients if the tumor lesions were not visible on MRI or if the image quality was poor as assessed by 2 radiologists (Q.W. and J.F.) with more than 9 years' experience and blinded to all clinical information. The recruitment pathway is shown in eFigure 1 in the Supplement.

After surgery, patients from Sun Yat-sen University Cancer Center and Yunnan Cancer Hospital were followed up with MRI or positron emission tomographic and computed tomographic imaging every 3 to 4 months for the first 2 years, every 6 months from the third to fifth years, and then annually. The end point of this study was DFS, which was defined as the period from the date of the operation to the date of the first local-regional recurrence, distant metastasis, all-cause mortality, or the latest follow-up used for censoring. Local-regional recurrences and distant metastasis were confirmed by gynecologic examination; imaging modalities, such as computed tomographic imaging, MRI, and positron emission tomographic and computed tomographic imaging; or biopsy findings.

### Image Acquisition and Preprocessing

All patients underwent pelvic MRI scans, including sagittal contrast-enhanced T1-weighted imaging (CET1WI), axial T2-weighted imaging (T2WI), and axial diffusion-weighted imaging (DWI). Magnetic resonance imaging scanning parameters are described in eMethods 1 in the Supplement. We generated apparent diffusion coefficient (ADC) maps to analyze DWI sequence (b values, 0 and 800 s/mm^2^).

**Figure 1.**
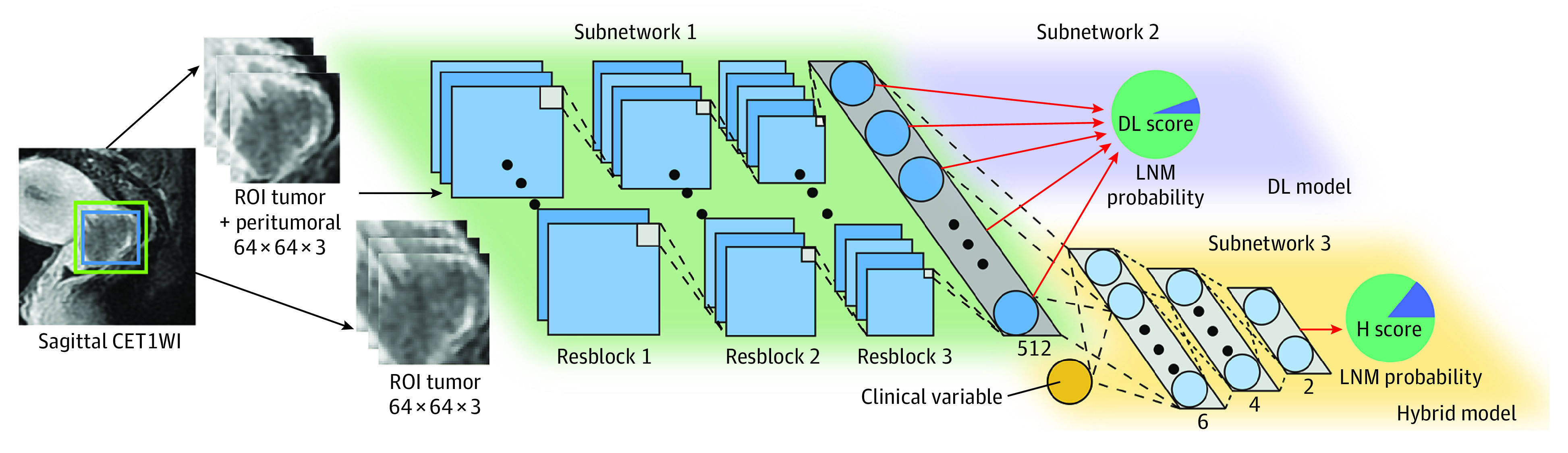
Illustration of the DL Model and the Hybrid Model The blue box on sagittal contrast-enhanced T1-weighted imaging (CET1WI) is a region of interest (ROI) tumor (tightly encapsulated tumor); the green box on sagittal CET1WI is an ROI tumor + peritumoral (5 pixels larger than the ROI tumor). Every 3 adjacent magnetic resonance imaging (MRI) sections were combined and scaled to 64 × 64 voxel size for deep learning (DL) analysis. The DL model consists of subnetworks 1 and 2, which are the stack of multiple convolutions, batch normalization, zero padding, and pooling layers. Feeding a tumor image, the DL model predicts the lymph node metastasis (LNM) probability (defined as DL score). The hybrid model consists of subnetworks 1 and 3, which integrate with the clinical variable (MRI-LN status). Feeding tumor images and the MRI-LN status of a patient, the hybrid model predicts the LNM probability at the end of subnetwork 3 (defined as H score).

To extract tumor information for analysis, the same 2 radiologists (Q.W. and J.F.) used rectangular bounding boxes for the region of interest (ROI) to tightly encapsulate tumors on MRI. This tight ROI was defined as *ROI tumor*. Because peritumoral regions were reported to have diagnostic value in predicting LN status,^13^ we also expanded ROI tumor by 5 pixels to add peritumoral information, defined as *ROI tumor + peritumoral*. Examples of ROI tumor and ROI tumor + peritumoral are shown in Figure 1.

### Model Development and Visualization

We developed an end-to-end DL model for LNM prediction (subnetworks 1 and 2 in Figure 1). The network was the stack of multiple convolutions, zero padding, and batch normalization layers. Layers were basic computational units in DL models,^30^ and the links of layers were similar to connections between neurons in brains; details of the layers are presented in eMethods 2 in the Supplement. Subnetwork 1 was similar to ResNet18, a widely used deep learning model,^31,32,33^ and the detailed network architecture is described in eMethods 3 and eFigure 2 in the Supplement. To enhance model training, subnetwork 1 was pretrained by 14 million natural images from the ImageNet data set^34,35^ and was fine-tuned using images from the primary cohort that comprised 5280 CET1WI, 1633 T2WI, and 1474 ADC map image sections. When an MR image of the tumor was fed into the DL model, subnetwork 2 predicted the LNM probability for the tumor. We defined the DL model–predicted LNM probability as the DL score. Owing to the inconsistency of previous research about the performance of MRI sequences,^13,14,15,16^ we compared the DL model among 3 MRI sequences to find the optimal model for LNM prediction.

As some preoperative clinical characteristics of cervical cancer have been reported to be associated with LNM,^36^ we evaluated 3 preoperative clinical factors (age, FIGO stage, and MRI-LN status) and selected the significant factors (*P* < .05) in the primary cohort to build clinical models. Because the DL model can mine high-dimensional information from MRI and clinical features can reflect tumor information from clinicopathologic aspects, we developed a hybrid model to combine information from these sources to explore whether they can be complementary (subnetworks 1 and 3 in Figure 1). We defined the hybrid model–predicted LNM probability as the H score. Detailed training processes of the DL and hybrid models are described in eMethods 4 in the Supplement.

To gain further intuition and explore the underlying basis of the end-to-end DL model, we applied visualization algorithms to display how the network learned the LNM-related information (eMethods 5 in the Supplement).^37^ We evaluated the DL model using the following methods: (1) visually assessing the area in the tumor that drew the attention of the DL model (defined as attention map), (2) visualizing convolutional features learned by the network (defined as DL feature), and (3) exploring the association between the DL feature and LN status. A discriminative DL feature should have different responses between patients with node-negative and node-positive findings.

### Statistical Analysis

All statistical analyses were performed with R, version 3.5.1 software (R Project for Statistical Computing). The statistical difference of clinical variables was assessed with an unpaired, 2-tailed χ^2^ test for categorical variables or *t* test for continuous variables. The Mann-Whitney test was applied to assess the difference of the DL score between patients with node-negative and node-positive findings. The DeLong test was applied to assess the difference of the receiver operating characteristic curves between different models.^38^ The Kaplan-Meier method and 2-sided log-rank tests were applied to estimate DFS. *P* < .05 indicated a statistically significant difference.

## Results

We reviewed 894 patients with stage IB to IIB cervical cancer who underwent radical hysterectomy and pelvic lymphadenectomy; 479 patients fulfilled the eligibility criteria and were enrolled in the primary (n = 338) and validation (n = 141) cohorts. The mean (SD) age of the patients was 49.1 (9.7) years. A total of 71 patients (21.0%) in the primary cohort and 32 patients (22.7%) in the validation cohort had LNM confirmed by lymphadenectomy (Table 1). As of December 2017, 188 patients from Sun Yat-sen University Cancer Center (30 lost to follow-up) and 128 patients from Yunnan Cancer Hospital (13 lost to follow-up) had completed the DFS follow-up.

**Table 1.** Characteristics of Patients in the Primary and Validation Cohorts

Characteristic	Primary cohort (n = 338)		*P* value^a^	Validation cohort (n = 141)		*P* value^a^	*P* value^b^
	No LNM	LNM		No LNM	LNM		
Patients, No. (%)	267 (79.0)	71 (21.0)		109 (77.3)	32 (22.7)		.77
Age, mean (SD), y	49.9 (9.5)	48.8 (10.0)	.40	48.0 (10.2)	47.6 (9.1)	.84	.07
FIGO stage, No. (%)^c^							
IB	145 (54.3)	28 (39.4)	<.001	81 (74.3)	22 (68.8)	.68	<.001
IIA	108 (40.4)	29 (40.8)		23 (21.1)	9 (28.1)		
IIB	14 (5.2)	14 (19.7)		5 (4.6)	1 (3.1)		
Differentiation grade, No. (%)							
Low	139 (52.1)	43 (60.6)	.44	51 (46.8)	19 (59.4)	.37	.65
Middle	124 (46.4)	27 (38.0)		56 (51.4)	12 (37.5)		
High	4 (1.5)	1 (1.4)		2 (1.8)	1 (3.1)		
MRI-LN status, No. (%)							
Negative	252 (94.4)	45 (63.4)	<.001	103 (94.5)	25 (78.1)	.01	.45
Positive	15 (5.6)	26 (36.6)		6 (5.5)	7 (21.9)		
Histologic characteristic, No. (%)							
Squamous cell carcinoma	225 (84.3)	61 (85.9)	.96	94 (86.2)	28 (87.5)	.91	.73
Adenocarcinoma	31 (11.6)	8 (11.3)		12 (11.0)	3 (9.4)		
Adenosquamous carcinoma	6 (2.2)	1 (1.4)		1 (0.9)	0		
Small cell carcinoma	5 (1.9)	1 (1.4)		2 (1.8)	1 (3.1)		
LVSI, No. (%)							
Negative	185 (69.3)	28 (39.4)	<.001	96 (88.1)	22 (68.8)	.02	<.001
Positive	82 (30.7)	43 (60.6)		13 (11.9)	10 (31.2)		

Abbreviations: FIGO, International Federation of Gynaecology and Obstetrics; LNM, lymph node metastasis; LVSI, lymphovascular invasion; MRI-LN, magnetic resonance imaging–reported lymph node.

^a^ *P* values were derived from the univariable association analyses of each clinicopathologic variable between patients with and without LNM in the primary and validation cohort.

^b^ *P* values represent the difference of each clinicopathologic variable between the primary and validation cohorts.

^c^ 2009 FIGO staging.^39^

### Diagnostic Performance of the Models

The MRI-LN status exhibited specificity of 94.38% in the primary cohort and 94.50% in the validation cohort, and sensitivity of 36.62% in the primary cohort and 21.88% in the validation cohort. The clinical model, which incorporated FIGO stage and MRI-LN status, yielded area under the curve (AUC) values of 0.704 (95% CI, 0.633-0.776) in the primary cohort and 0.622 (95% CI, 0.519-0.725) in the validation cohort (Table 2).

**Table 2.** Diagnostic Performance of Various Models

Model	Primary cohort, % (95% CI)				Validation cohort, % (95% CI)			
	AUC	Accuracy	Sensitivity	Specificity	AUC	Accuracy	Sensitivity	Specificity
**Clinical **									
MRI-LN status	0.655 (0.597-0.713)	82.25 (77.75-86.17)	36.62 (25.75-48.95)	94.38 (90.71-96.71)	0.582 (0.506-0.658)	78.01 (70.27-84.55)	21.88 (9.94-40.44)	94.50 (87.92-97.74)^a^
FIGO stage	0.604 (0.532-0.674)	55.62 (50.15-61.00)	60.56 (48.23-71.74)	54.31 (48.13-60.36)	0.525 (0.434-0.616)	64.54 (56.05-72.41)	31.25 (16.75-50.14)	74.31 (64.89-81.99)
MRI-LN status + FIGO stage	0.704 (0.633-0.776)	80.47 (75.84-84.56)	45.07 (33.40-57.28)	89.89 (85.47-93.11)	0.622 (0.519-0.725)	66.67 (58.24-74.37)	50.00 (32.24-67.76)	71.56 (61.99-79.59)
**Deep learning**									
CET1WI tumor + peritumoral	0.894 (0.857-0.931)	75.15 (70.18-79.66)	88.73 (78.47-94.66)	71.54 (65.65-76.79)	0.844 (0.780-0.907)	74.47 (66.45-81.43)	87.50 (70.07-95.92)	70.64 (61.03-78.78)
CET1WI tumor	0.845 (0.794-0.896)	76.92 (72.06-81.31)	78.87 (67.25-87.32)	76.40 (70.76-81.27)	0.742 (0.651-0.833)	60.99 (52.43-69.09)	81.25 (62.96-92.14)	55.05 (45.24-64.49)
T2WI tumor + peritumoral	0.671 (0.601-0.742)	56.51 (51.04-61.86)	78.87 (67.25-87.32)	50.56 (44.41-56.69)	0.651 (0.540-0.762)	78.72 (71.04-85.16)	37.50 (21.66-56.25)	90.83 (83.38-95.27)
ADC tumor + peritumoral	0.702 (0.634-0.770)	71.01 (65.85-75.79)	59.15 (46.84-70.47)	74.16 (68.39-79.21)	0.667 (0.563-0.770)	58.87 (50.27-67.08)	78.12 (59.56-90.06)	53.21 (43.45-62.75)
**Hybrid **									
CET1WI tumor + peritumoral + MRI-LN status	0.963 (0.930-0.996)^a^	96.45 (93.88-98.15)^a^	92.96 (83.65-97.38)^a^	97.38 (94.44-98.85)^a^	0.933 (0.887-0.979)^a^	87.94 (81.40-92.82)^a^	90.62 (73.83-97.55)^a^	87.16 (79.06-92.55)

Abbreviations: ADC, apparent diffusion coefficient; AUC, area under the receiver operating characteristic curve; CET1WI, contrast-enhanced T1-weighted imaging; FIGO, International Federation of Gynaecology and Obstetrics; MRI-LN, magnetic resonance imaging–reported lymph node; T2WI, T2-weighted imaging.

^a^ Best performance.

**Figure 2.**
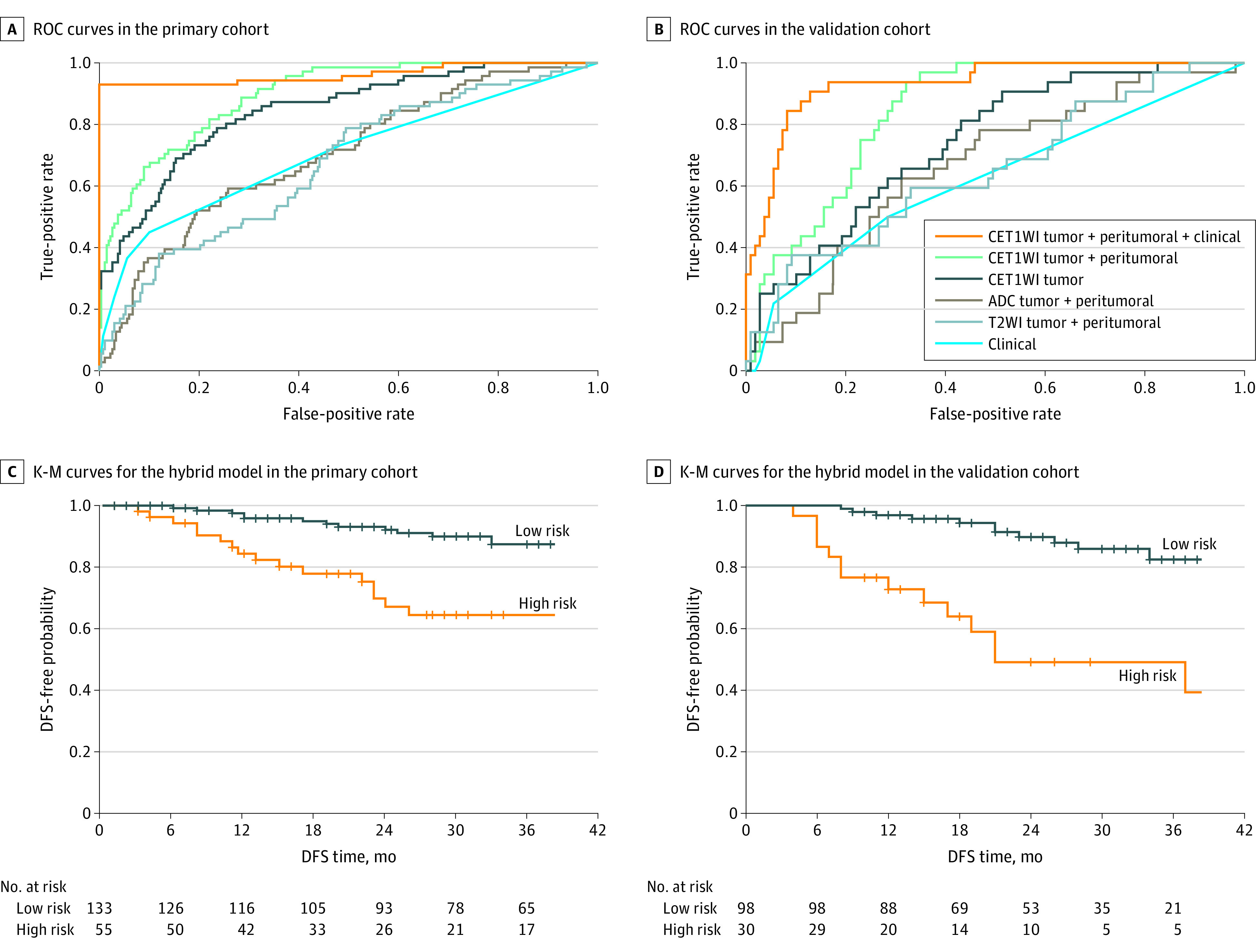
Performance of Various Models Receiver operating characteristic (ROC) curves in the primary (A) and validation (B) cohorts of the contrast-enhanced T1-weighted imaging (CET1WI) tumor + peritumoral + clinical, CET1WI tumor + peritumoral, CET1WI tumor, apparent diffusion coefficient (ADC) tumor + peritumoral, T2-weighted imaging (T2WI) tumor + peritumoral, and clinical model. Survival curves according to the H score from the hybrid model with Kaplan-Meier (K-M) analysis in the primary (C) and validation (D) cohorts. DFS indicates disease-free survival.

Among all the DL models (Figure 2A,B), the CET1WI tumor + peritumoral illustrated the best performance in detecting metastatic LN in both the primary cohort (AUC, 0.894; 95% CI, 0.857-0.931) and validation cohort (AUC, 0.844; 95% CI, 0.780-0.907). The DL score determined from CET1WI tumor + peritumoral revealed a significant difference between patients with node-positive and node-negative findings in both the primary (0.58; interquartile range [IQR], 0.46-0.67 vs 0.34; IQR, 0.27-0.43; *P* < .001) and validation (0.47; IQR, 0.43-0.56 vs 0.35; IQR, 0.27-0.43; *P* < .001) cohorts (eFigure 3A in the Supplement). We found that the DL model using both intratumoral and peritumoral regions (CET1WI tumor + peritumoral) outperformed the model using only intratumoral regions (CET1WI tumor) in the primary (AUC, 0.894; 95% CI, 0.857-0.932 vs AUC, 0.845; 95% CI, 0.794-0.896; *P* = .006) and validation (AUC, 0.844; 95% CI, 0.780-0.907 vs AUC, 0.742; 95% CI, 0.651-0.833; *P* = .006) cohorts (Table 2).

To further assess the added value of the DL model to the MRI-LN status, we conducted stratified analysis within MRI-LN subgroups. Within the negative MRI-LN subgroup, the DL score achieved an AUC of 0.877 (95% CI, 0.828-0.926) in the primary cohort and 0.841 (95% CI, 0.772-0.911) in the validation cohort. Within the positive MRI-LN subgroup, the AUC was 0.956 (95% CI, 0.893-1.000) in the primary cohort and 0.905 (95% CI, 0.707-1.000) in the validation cohort. Moreover, the DL score exhibited a significant difference between patients with node-positive and node-negative findings in the primary cohort (DL score among MRI-LN-positive patients: node-positive vs node-negative, 0.60; IQR, 0.52-0.67 vs 0.29; IQR, 0.26-0.36; *P* < .001; DL score among MRI-LN-negative patients: node-positive vs node-negative, 0.56; IQR, 0.45-0.67 vs 0.35; IQR, 0.27-0.43; *P* < .001) and validation cohort (DL score among MRI-LN-positive patients: node-positive vs node-negative, 0.45; IQR, 0.43-0.56 vs 0.35; IQR, 0.29-0.38; *P* < .001; DL score among MRI-LN-negative patients: node-positive vs node-negative, 0.47; IQR, 0.44-0.56 vs 0.35; IQR, 0.27-0.43; *P* < .001) (eFigure 3B in the Supplement).

**Figure 3.**
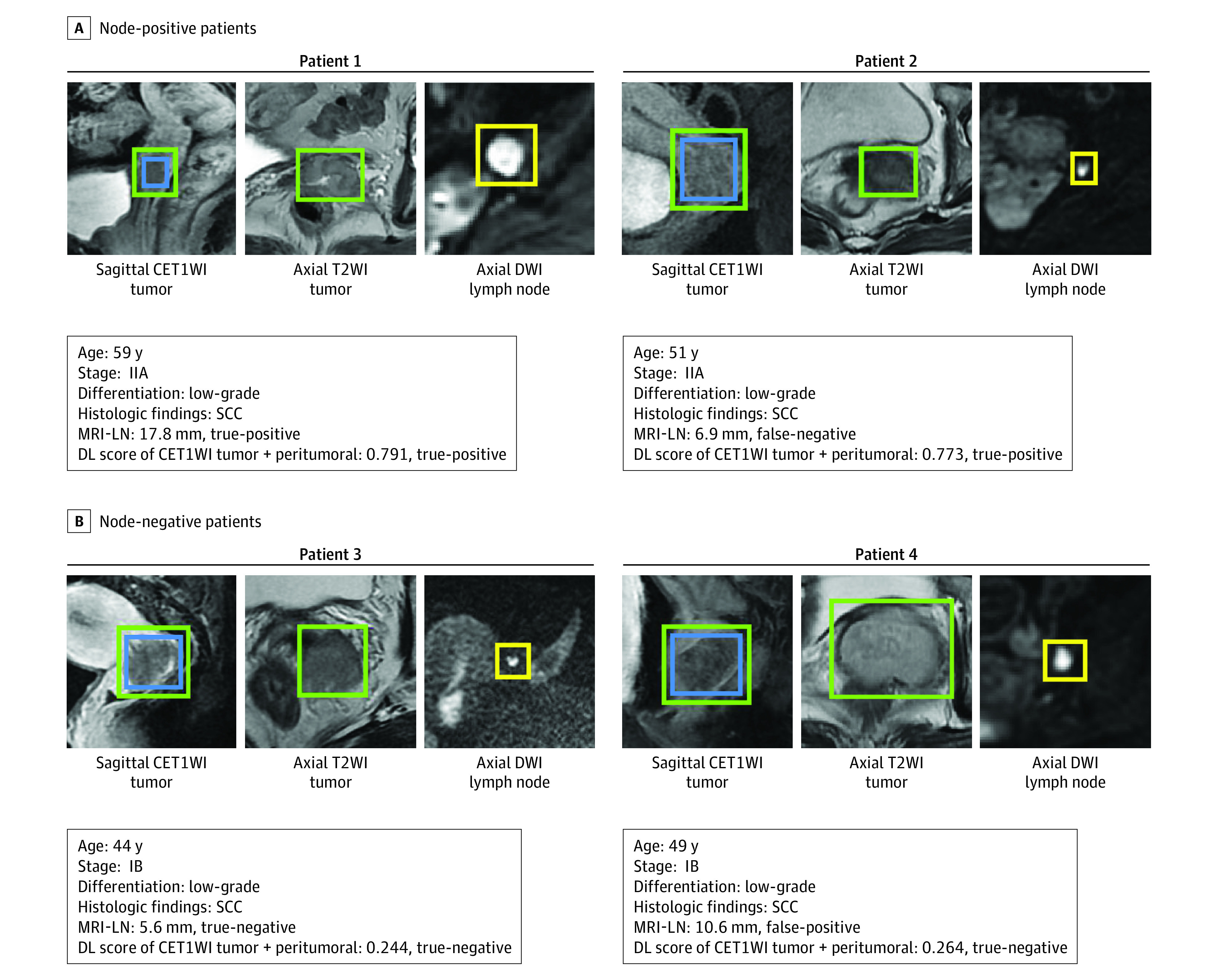
Representative Prediction Results From the Validation Cohort The blue boxes on sagittal contrast-enhanced T1-weighted imaging (CET1WI) are region of interest (ROI) tumor, the green boxes on sagittal CET1WI and axial T2-weighted imaging (T2WI) are ROI tumor + peritumoral, and the yellow boxes on axial diffusion-weighted imaging (DWI) are lymph nodes. Positive magnetic resonance imaging (MRI)-reported lymph node (MRI-LN) status was assessed by the short-axis diameter of the largest lymph node larger than 10 mm. DL indicates deep learning; SCC, squamous cell carcinoma.

To further illustrate the predictive performance of the DL model, we depicted 4 representative prediction results in Figure 3. The 4 patients had similar clinicopathologic characteristics, making it difficult to identify LN status by clinical characteristics and visual observation on MRI. However, the DL model was able to generate discriminative predictive value.

Because the DL model of CET1WI tumor + peritumoral exhibited the highest sensitivity and MRI-LN status exhibited the highest specificity, a combined hybrid model was established. The hybrid model showed significant improvement either in the primary cohort (AUC, 0.963; 95% CI, 0.930-0.996 vs AUC, 0.894; 95% CI, 0.857-0.931; *P* < .001) and validation cohort (AUC, 0.933; 95% CI, 0.887-0.979 vs AUC, 0.844; 95% CI, 0.780-0.907; *P* = .008). The hybrid model achieved AUC, 0.963; sensitivity, 92.96%; and specificity, 97.38% in the primary cohort and AUC, 0.933; sensitivity, 90.62%; and specificity, 87.16% in the validation cohort.

Assisted by the DL visualization algorithms, we discovered a high-response area for each tumor (eFigure 4 in the Supplement). These high-response areas were more important than other parts of tumors because they drew more attention to the DL model and consequently contained more LNM-related information. These high-response areas included both intratumoral and peritumoral areas, indicating that both intratumoral and peritumoral regions were necessary for the DL model to make decisions.

To have a better understanding of the DL feature learned by the network, we visualized representative DL features from convolution layers (eFigure 5A in the Supplement). In the shallow convolution layers, the DL model extracted simple tumor edge features (the second and sixth layers), while in deeper convolution layers, it extracted complex tumor texture information (the tenth layer). In the last convolution layer, the DL model extracted high-level abstract features (the fourteenth layer). Although these high-level features were so intricate that they were hard to interpret by general gross observation, they were associated with LN status. As shown in eFigure 5B in the Supplement, the patients with node-negative findings had weaker DL-feature responses and vice versa, indicating that the network learned discriminative DL features for LNM prediction.

In eFigure 6A in the Supplement, we visualized 2 DL features of the last convolution layer to explore the association between DL features and LNM. The positive DL feature had strong responses to patients with node-positive findings and weak responses to those with node-negative findings. Similarly, the negative DL feature had strong responses to patients free of LNM and was nearly shut down in patients with LNM. The response value of negative and positive DL features also showed a statistically significant difference between patients with node-positive and node-negative findings in the primary (DL feature response among positive DL feature status: node-positive vs node-negative, −0.014; IQR, −0.104 to 0.077 vs −0.037; IQR, −0.126 to 0.048; *P* < .001; DL feature response among negative DL feature status: node-positive vs node-negative, −0.195; IQR, −0.291 to −0.114 vs −0.176; IQR, −0.259 to −0.095; *P* < .001) and validation (DL feature response among positive DL feature status: node-positive vs node-negative, 0.030; IQR, −0.059 to 0.111 vs −0.118, IQR, −0.096 to 0.076; *P* < .001; DL feature response among negative DL feature status: node-positive vs node-negative, −0.182; IQR, −0.257 to −0.103 vs −0.146; IQR, −0.216 to −0.078; *P* < .001) cohorts (eFigure 6B in the Supplement). These results suggest that the DL feature is discriminative in diagnosing LNM.

### Prognostic Value of the Hybrid Model 

Because the LN status of cervical cancer has been reported to be a crucial prognostic factor,^40,41^ we performed survival analyses to assess the prognostic ability of the hybrid model with regard to DFS. We used the median H score to stratify patients into low- and high-risk groups.

The median survival time for DFS was 31 (IQR, 16-56) months in the primary cohort and 23 (IQR, 14-33) months in the validation cohort. Figure 2C, D shows a significant difference between low- and high-risk patients from the hybrid model in the primary cohort (hazard ratio, 3.24; 95% CI, 1.64-6.44; *P* < .001) and validation cohort (hazard ratio, 4.59; 95% CI, 2.04-10.31; *P* < .001). Patients with higher H scores had a shorter time to reach the DFS.

## Discussion

In this multicenter study, we developed an end-to-end DL model to diagnose LNM for patients with cervical cancer preoperatively. We compared the DL model among different MRI sequences (CET1WI, T2WI, and DWI) and explored the diagnostic value of intratumoral and peritumoral regions. Among all DL models, the CET1WI tumor + peritumoral model achieved the best performance, indicating that the CET1WI sequence probably contained more LNM-related information than the other 2 sequences (T2WI and DWI). To mine diagnostic information from both MR images and clinical characteristics, a hybrid model combining the CET1WI tumor + peritumoral model with MRI-LN status was established. This hybrid model appears to be able to identify more than 90% of metastatic LN cases with a specificity of more than 87%. Moreover, we found that the H score was significantly associated with DFS of cervical cancer, indicating that the hybrid model was a good prognostic indicator.

In previous studies, peritumoral regions in cervical cancer have been shown to be valuable in diagnosing LNM and estimating neoadjuvant chemotherapy response.^13,42^ Therefore, we compared the 2 DL models using ROI tumor + peritumoral and ROI tumor. Contrary to CET1WI tumor + peritumoral, the AUC of the CET1WI tumor decreased from 0.844 to 0.742, suggesting that peritumoral regions played a role in predicting LNM in cervical cancer. Adding peritumoral regions led to increased AUC, which can probably be explained by the fact that higher lymphatic vessel density in peritumoral regions might lead to higher regional LNM.^43^ As reported in previous studies, an increase in lymphatic vessel density can change the tumor microenvironment and metastatic propensity,^44^ which is reflected in many cancers, including cervical, prostate, and breast cancer.^43,45,46^ Findings shown in eFigure 4 in the Supplement suggest that the DL model also used both intratumoral and peritumoral regions to make its final decision.

Owing to the high sensitivity of the CET1WI tumor + peritumoral model and the high specificity of MRI-LN status, we developed a hybrid model to integrate image-level and clinicopathologic-level information, resulting in an increase in the AUC from 0.844 to 0.933, sensitivity from 87.5% to 90.62%, and specificity from 70.64% to 87.16%. These improvements suggest that the DL model mined complementary information to the MRI-LN status. Therefore, with the apparent high sensitivity and specificity of our hybrid model, this model might be used preoperatively to help gynecologists make decisions.

In clinical practice, the following 2 scenarios may result in an inappropriate treatment plan: lymphadenopathy not detected on MRI but positive results shown in surgery (patient 2 in Figure 3) and lymphadenopathy detected on MRI but proved to be negative (patient 4 in Figure 3). Therefore, we applied stratified analysis to explore the added value of the DL model within MRI-LN subgroups. As shown in eFigure 3B in the Supplement, the DL score from the CET1WI tumor + peritumoral model exhibited a significant difference between patients with node-positive and node-negative findings within MRI-LN subgroups in the primary and validation cohorts (all *P* < .001). Therefore, the DL model may benefit patients with false-negative and false-positive LN status on routine MRI.

In contrast with previous studies, our study develops an end-to-end DL model to detect LNM during routine MRI. Attempts have been made to assess LN status, such as sentinel nodes biopsy, applying clinical factors, and radiomic analysis. Although sentinel LN dissection as an invasive method shows good sensitivity and specificity,^47^ its application is limited by available facilities and experts.^48,49,50^ The sensitivity of clinical characteristics (eg, FIGO stage and MRI-LN status) is not sufficient to help inform decision-making by clinicians. Radiomic analysis requires time-consuming tumor delineation, which affects the reproducibility of radiomic features.^51^ Although radiomic features can reflect some generalized image features, those characteristics might not be adaptive to LNM prediction. Consequently, we developed a DL model to try to overcome these problems by automatically learning LNM-related features, providing a helpful adjunct to assess LNM.

### Limitations

Despite the favorable diagnostic performance of the DL model, our research has limitations. First, a more extensive and prospective data set is needed to generalize the performance of the DL model. Second, although CET1WI showed better performance than T2WI and ADC maps, the combination of these sequences is unclear.

## Conclusions

The findings of this study suggest that DL may serve as a preoperative noninvasive tool to diagnose LNM in women with cervical cancer. The H score from the hybrid model was significantly associated with the prognosis of cervical cancer.

## Notes

Supplement. **eMethods 1.** Magnetic Resonance Image Acquisition Parameters Used in the Present Study **eMethods 2.** Mathematical Description of the Deep Learning Network **eMethods 3.** Development of the DL Model and the Hybrid Model **eMethods 4.** Training Process of the DL Model and the Hybrid Model **eMethods 5.** Details of the DL Model Visualization **eFigure 1.** Patient Flowchart **eFigure 2.** Architecture of the DL and Hybrid Model **eFigure 3.** Performance of the DL Score **eFigure 4.** Response Area of Representative Patients **eFigure 5.** The DL-Feature Visualization **eFigure 6.** The DL-Feature Analysis **eReferences.**

## References

[1] Siegel RL, Miller KD, Jemal A Cancer statistics, 2019. CA Cancer J Clin. 2019;69(1):7-34. doi:10.3322/caac.21551 30620402

[2] Bhatla N, Denny L FIGO cancer report 2018. Int J Gynaecol Obstet. 2018;143(suppl 2):2-3. doi:10.1002/ijgo.12608 30306587

[3] Matsuo K, Machida H, Mandelbaum RS, Konishi I, Mikami M Validation of the 2018 FIGO cervical cancer staging system. Gynecol Oncol. 2019;152(1):87-93. doi:10.1016/j.ygyno.2018.10.026 30389105 PMC7528458

[4] Cheng X, Cai S, Li Z, Tang M, Xue M, Zang R The prognosis of women with stage IB1-IIB node-positive cervical carcinoma after radical surgery. World J Surg Oncol. 2004;2(1):47-48. doi:10.1186/1477-7819-2-47 15606922 PMC546224

[5] Tsai CS, Lai CH, Wang CC, The prognostic factors for patients with early cervical cancer treated by radical hysterectomy and postoperative radiotherapy. Gynecol Oncol. 1999;75(3):328-333. doi:10.1006/gyno.1999.5527 10600284

[6] Kokka F, Bryant A, Brockbank E, Powell M, Oram D Hysterectomy with radiotherapy or chemotherapy or both for women with locally advanced cervical cancer. Cochrane Database Syst Rev. 2015;20(4):CD010260. doi:10.1002/14651858.CD010260.pub2 25847525

[7] Matsuura Y, Kawagoe T, Toki N, Tanaka M, Kashimura M Long-standing complications after treatment for cancer of the uterine cervix—clinical significance of medical examination at 5 years after treatment. Int J Gynecol Cancer. 2006;16(1):294-297. doi:10.1111/j.1525-1438.2006.00354.x 16445648

[8] Landoni F, Maneo A, Colombo A, Randomised study of radical surgery versus radiotherapy for stage Ib-IIa cervical cancer. Lancet. 1997;350(9077):535-540. doi:10.1016/S0140-6736(97)02250-2 9284774

[9] Balcacer P, Shergill A, Litkouhi B MRI of cervical cancer with a surgical perspective: staging, prognostic implications and pitfalls. Abdom Radiol (NY). 2019;44(7):2557-2571. doi:10.1007/s00261-019-01984-7 30903231

[10] Choi HJ, Roh JW, Seo SS, Comparison of the accuracy of magnetic resonance imaging and positron emission tomography/computed tomography in the presurgical detection of lymph node metastases in patients with uterine cervical carcinoma: a prospective study. Cancer. 2006;106(4):914-922. doi:10.1002/cncr.21641 16411226

[11] McMahon CJ, Rofsky NM, Pedrosa I Lymphatic metastases from pelvic tumors: anatomic classification, characterization, and staging. Radiology. 2010;254(1):31-46. doi:10.1148/radiol.2541090361 20032141

[12] Chung HH, Kang KW, Cho JY, Role of magnetic resonance imaging and positron emission tomography/computed tomography in preoperative lymph node detection of uterine cervical cancer. Am J Obstet Gynecol. 2010;203(2):156.e1-156.e5. doi:10.1016/j.ajog.2010.02.041 20435285

[13] Wu Q, Wang S, Chen X, Radiomics analysis of magnetic resonance imaging improves diagnostic performance of lymph node metastasis in patients with cervical cancer. Radiother Oncol. 2019;138:141-148. doi:10.1016/j.radonc.2019.04.035 31252296

[14] Wang T, Gao T, Yang J, Preoperative prediction of pelvic lymph nodes metastasis in early-stage cervical cancer using radiomics nomogram developed based on T2-weighted MRI and diffusion-weighted imaging. Eur J Radiol. 2019;114:128-135. doi:10.1016/j.ejrad.2019.01.003 31005162

[15] Kan Y, Dong D, Zhang Y, Radiomic signature as a predictive factor for lymph node metastasis in early-stage cervical cancer. J Magn Reson Imaging. 2019;49(1):304-310. doi:10.1002/jmri.26209 30102438

[16] Yu YY, Zhang R, Dong RT, Feasibility of an ADC-based radiomics model for predicting pelvic lymph node metastases in patients with stage IB-IIA cervical squamous cell carcinoma. Br J Radiol. 2019;92(1097):20180986. doi:10.1259/bjr.20180986 30888846 PMC6580913

[17] Kermany DS, Goldbaum M, Cai W, Identifying medical diagnoses and treatable diseases by image-based deep learning. Cell. 2018;172(5):1122-1131.e9. doi:10.1016/j.cell.2018.02.010 29474911

[18] Wang L, Sha L, Lakin JR, Development and validation of a deep learning algorithm for mortality prediction in selecting patients with dementia for earlier palliative care interventions. JAMA Netw Open. 2019;2(7):e196972. doi:10.1001/jamanetworkopen.2019.6972 31298717 PMC6628612

[19] Park A, Chute C, Rajpurkar P, Deep learning–assisted diagnosis of cerebral aneurysms using the HeadXNet Model. JAMA Netw Open. 2019;2(6):e195600. doi:10.1001/jamanetworkopen.2019.5600 31173130 PMC6563570

[20] Ding Y, Sohn JH, Kawczynski MG, A deep learning model to predict a diagnosis of Alzheimer disease by using ^18^F-FDG PET of the brain. Radiology. 2019;290(2):456-464. doi:10.1148/radiol.2018180958 30398430 PMC6358051

[21] Yala A, Lehman C, Schuster T, Portnoi T, Barzilay R A deep learning mammography–based model for improved breast cancer risk prediction. Radiology. 2019;292(1):60-66. doi:10.1148/radiol.2019182716 31063083

[22] Hwang EJ, Park S, Jin KN, ; DLAD Development and Evaluation Group Development and validation of a deep learning–based automated detection algorithm for major thoracic diseases on chest radiographs. JAMA Netw Open. 2019;2(3):e191095. doi:10.1001/jamanetworkopen.2019.1095 30901052 PMC6583308

[23] Hu L, Bell D, Antani S, An observational study of deep learning and automated evaluation of cervical images for cancer screening. J Natl Cancer Inst. 2019;111(9):923-932. doi:10.1093/jnci/djy225 30629194 PMC6748814

[24] Valdes G, Interian Y Comment on “deep convolutional neural network with transfer learning for rectum toxicity prediction in cervical cancer radiotherapy: a feasibility study.” Phys Med Biol. 2018;63(6):068001. doi:10.1088/1361-6560/aaae23 29424369

[25] Liu Z, Wang S, Dong D, The applications of radiomics in precision diagnosis and treatment of oncology: opportunities and challenges. Theranostics. 2019;9(5):1303-1322. doi:10.7150/thno.30309 30867832 PMC6401507

[26] Parmar C, Barry JD, Hosny A, Quackenbush J, Aerts HJWL Data analysis strategies in medical imaging. Clin Cancer Res. 2018;24(15):3492-3499. doi:10.1158/1078-0432.CCR-18-0385 29581134 PMC6082690

[27] Wang S, Shi J, Ye Z, Predicting EGFR mutation status in lung adenocarcinoma on computed tomography image using deep learning. Eur Respir J. 2019;53(3):1800986. doi:10.1183/13993003.00986-2018 30635290 PMC6437603

[28] Wang S, Liu Z, Rong Y, Deep learning provides a new computed tomography–based prognostic biomarker for recurrence prediction in high-grade serous ovarian cancer. Radiother Oncol. 2019;132:171-177. doi:10.1016/j.radonc.2018.10.019 30392780

[29] Ardila D, Kiraly AP, Bharadwaj S, Author correction: end-to-end lung cancer screening with three-dimensional deep learning on low-dose chest computed tomography. Nat Med. 2019;25(8):1319. doi:10.1038/s41591-019-0536-x 31253948

[30] LeCun Y, Bengio Y, Hinton G Deep learning. Nature. 2015;521(7553):436-444. doi:10.1038/nature14539 26017442

[31] He K, Zhang X, Ren S, Sun J Deep residual learning for image recognition. Published December 10, 2015. Accessed April 27, 2020. https://arxiv.org/abs/1512.03385

[32] Chang K, Bai HX, Zhou H, Residual convolutional neural network for the determination of *IDH* status in low- and high-grade gliomas from MR imaging. Clin Cancer Res. 2018;24(5):1073-1081. doi:10.1158/1078-0432.CCR-17-2236 29167275 PMC6051535

[33] Wang S, Zhou M, Liu Z, Central focused convolutional neural networks: developing a data-driven model for lung nodule segmentation. Med Image Anal. 2017;40:172-183. doi:10.1016/j.media.2017.06.014 28688283 PMC5661888

[34] Deng J, Dong W, Socher R, Li L, Li K, Fei-Fei L ImageNet: a large-scale hierarchical image database. Accessed April 27, 2020. https://image-net.org/papers/imagenet_cvpr09.pdf

[35] Yosinski J, Clune J, Bengio Y, Lipson H How transferable are features in deep neural networks? Published November 6, 2014. Accessed April 27, 2020. https://arxiv.org/abs/1411.1792

[36] Kim DY, Shim SH, Kim SO, Preoperative nomogram for the identification of lymph node metastasis in early cervical cancer. Br J Cancer. 2014;110(1):34-41. doi:10.1038/bjc.2013.718 24231954 PMC3887306

[37] Selvaraju RR, Cogswell M, Das A, Vedantam R, Parikh D, Batra D Grad-CAM: visual explanations from deep networks via gradient-based localization. Updated December 3, 2019. Accessed April 27, 2020. https://arxiv.org/abs/1610.02391

[38] DeLong ER, DeLong DM, Clarke-Pearson DL Comparing the areas under two or more correlated receiver operating characteristic curves: a nonparametric approach. Biometrics. 1988;44(3):837-845. doi:10.2307/2531595 3203132

[39] Pecorelli S Revised FIGO staging for carcinoma of the vulva, cervix, and endometrium. Int J Gynaecol Obstet. 2009;105(2):103-104. doi:10.1016/j.ijgo.2009.02.012 19367689

[40] Taarnhøj GA, Christensen IJ, Lajer H, Risk of recurrence, prognosis, and follow-up for Danish women with cervical cancer in 2005-2013: a national cohort study. Cancer. 2018;124(5):943-951. doi:10.1002/cncr.31165 29211304

[41] Oaknin A, Rubio MJ, Redondo A, SEOM guidelines for cervical cancer. Clin Transl Oncol. 2015;17(12):1036-1042. doi:10.1007/s12094-015-1452-2 26650487 PMC4689764

[42] Sun C, Tian X, Liu Z, Radiomic analysis for pretreatment prediction of response to neoadjuvant chemotherapy in locally advanced cervical cancer: a multicentre study. EBioMedicine. 2019;46:160-169. doi:10.1016/j.ebiom.2019.07.049 31395503 PMC6712288

[43] Gombos Z, Xu X, Chu CS, Zhang PJ, Acs G Peritumoral lymphatic vessel density and vascular endothelial growth factor C expression in early-stage squamous cell carcinoma of the uterine cervix. Clin Cancer Res. 2005;11(23):8364-8371. doi:10.1158/1078-0432.CCR-05-1238 16322297

[44] Botting SK, Fouad H, Elwell K, Prognostic significance of peritumoral lymphatic vessel density and vascular endothelial growth factor receptor 3 in invasive squamous cell cervical cancer. Transl Oncol. 2010;3(3):170-175. doi:10.1593/tlo.09292 20563258 PMC2887646

[45] El-Gohary YM, Metwally G, Saad RS, Robinson MJ, Mesko T, Poppiti RJ Prognostic significance of intratumoral and peritumoral lymphatic density and blood vessel density in invasive breast carcinomas. Am J Clin Pathol. 2008;129(4):578-586. doi:10.1309/2HGNJ1GU57JMBJAQ 18343785

[46] Roma AA, Magi-Galluzzi C, Kral MA, Jin TT, Klein EA, Zhou M Peritumoral lymphatic invasion is associated with regional lymph node metastases in prostate adenocarcinoma. Mod Pathol. 2006;19(3):392-398. doi:10.1038/modpathol.3800546 16400321

[47] Salvo G, Ramirez PT, Levenback CF, Sensitivity and negative predictive value for sentinel lymph node biopsy in women with early-stage cervical cancer. Gynecol Oncol. 2017;145(1):96-101. doi:10.1016/j.ygyno.2017.02.005 28188015 PMC5873580

[48] Bhatla N, Berek JS, Cuello Fredes M, Revised FIGO staging for carcinoma of the cervix uteri. Int J Gynaecol Obstet. 2019;145(1):129-135. doi:10.1002/ijgo.12749 30656645

[49] Lécuru F, Mathevet P, Querleu D, Bilateral negative sentinel nodes accurately predict absence of lymph node metastasis in early cervical cancer: results of the SENTICOL study. J Clin Oncol. 2011;29(13):1686-1691. doi:10.1200/JCO.2010.32.0432 21444878

[50] Ferrandina G, Pedone Anchora L, Gallotta V, Can we define the risk of lymph node metastasis in early-stage cervical cancer patients? a large-scale, retrospective study. Ann Surg Oncol. 2017;24(8):2311-2318. doi:10.1245/s10434-017-5917-0 28608117

[51] Fiset S, Welch ML, Weiss J, Repeatability and reproducibility of MRI-based radiomic features in cervical cancer. Radiother Oncol. 2019;135:107-114. doi:10.1016/j.radonc.2019.03.001 31015155

