# Supplementary material for: Development of a Deep Learning Model to Identify Lymph Node Metastasis on Magnetic Resonance Imaging in Patients With Cervical Cancer
Source: JAMA Netw Open. 2020 Jul 24;3(7):e2011625. doi: 10.1001/jamanetworkopen.2020.11625 (PMC7382006; doi:10.1001/jamanetworkopen.2020.11625)
Supplement: Supplement. — eMethods 1. Magnetic Resonance Image Acquisition Parameters Used in the Present Study eMethods 2. Mathematical Description of the Deep Learning Network eMethods 3. Development of the DL Model and the Hybrid Model eMethods 4. Training Process of the DL Model and the Hybrid Model eMethods 5. Details of the DL Model Visualization eFigure 1. Patient Flowchart eFigure 2. Architecture of the DL and Hybrid Model eFigure 3. Performance of the DL Score eFigure 4. Response Area of Representative Patients eFigure 5. The DL-Feature Visualization eFigure 6. The DL-Feature Analysis eReferences. [file jamanetwopen-3-e2011625-s001.pdf]

Supplementary Online Content

Wu Q, Wang S, Fang J, et al. Development of a deep learning model to identify lymph node metastasis on magnetic resonance imaging in patients with cervical cancer. *JAMA Netw Open*. 2020;3(7):e2011625. doi:10.1001/jamanetworkopen.2020.11625

eMethods 1. Magnetic Resonance Image Acquisition Parameters Used in the Present Study

eMethods 2. Mathematical Description of the Deep Learning Network

eMethods 3. Development of the DL Model and the Hybrid Model

eMethods 4. Training Process of the DL Model and the Hybrid Model

eMethods 5. Details of the DL Model Visualization

eFigure 1. Patient Flowchart

eFigure 2. Architecture of the DL and Hybrid Model

eFigure 3. Performance of the DL Score

eFigure 4. Response Area of Representative Patients

eFigure 5. The DL-Feature Visualization

eFigure 6. The DL-Feature Analysis

eReferences

This supplementary material has been provided by the authors to give readers additional information about their work.

© 2020 Wu Q et al. *JAMA Network Open.*

eMethods 1. Magnetic resonance image acquisition parameters used in the present study.

Yunnan Cancer Hospital: All the patients from the Yunnan Cancer Hospital were examined using the SIEMENS 1.5T Avanto MRI with the following scanning parameters: axial T2-weighted spin-echo images (repetition time [TR]/ echo time [TE]: 4000/100 ms, field of view [FOV] = 20 × 18 cm, number of excitation [NEX] = 4, slice thickness = 3 mm, spacing between slices = 0.3 mm) and sagittal contrast-enhanced T1-weighted spin-echo images (TR/TE: 4.65/1.55 ms, FOV = 26 × 22 cm, NEX = 8, slice thickness = 3.6 mm, spacing between slices = 0.7 mm). Axial DWI (TR/TE: 6000/55 ms; FOV: 200 × 180 mm^2; matrix: 256 × 256; slice thickness/gap: 3/0.4 mm; b values of 0 and 800 s/mm^2) were obtained by using single-shot spin-echo echo-planar imaging (EPI).

Sun Yat-sen University Cancer Center: The patients from Sun Yat-sen University Cancer Center were examined using the 3.0T GE Discovery750 MRI and the 1.5T GE Signa MRI.

The 3.0T GE Discovery750 MRI acquisition parameters were as follows: axial T2-weighted spin-echo images (TR/TE: 3966/86 ms, FOV = 36 × 36 cm, NEX = 2, slice thickness = 5 mm, spacing between slices = 1mm) and sagittal contrast-enhanced T1-weighted spin-echo images (TR/TE: 5.25/1.82 ms, FOV = 28× 28 cm, NEX = 1, slice thickness = 3 mm, spacing between slices = 0 mm). Axial DWI (TR/TE: 4800/75 ms; FOV: 240 × 200 mm^2; matrix: 160 × 112; slice thickness/gap: 3/0.3 mm; b values of 0 and 800 s/mm^2) were obtained by using single-shot spin-echo EPI.

The 1.5T GE Signa MRI acquisition parameters were as follows: axial T2-weighted spin-echo images (TR/TE: 3283/87 ms, FOV = 36 × 36 cm, NEX = 2, slice thickness =5 mm, spacing between slices = 1 mm) and sagittal contrast-enhanced T1-weighted spin-echo images (TR/TE: 4.1/1.96 ms, FOV = 28× 26 cm, NEX = 1, slice thickness = 3 mm, spacing between slices = 0 mm). Axial DWI (TR/TE: 4721/87 ms; FOV: 260 × 220 mm^2; matrix: 160 × 112; slice thickness/ gap: 3/0.3 mm; b values of 0 and 800 s/mm^2) were obtained by using single-shot spin-echo EPI.

Henan Provincial People's Hospital: The patients from Henan Provincial People’s Hospital underwent pelvic MRI using one of the two 3.0-T MR image systems, either with (Discovery MR 750; GE Medical Systems, Milwaukee, Wis) or (Magnetom TrioTim; Siemens Healthineers) equipped with an 8-channel phased-array coil in supine position. Axial T2-weighted spin-echo images (TR/TE: 4000/85 ms, FOV = 34 × 34 cm, NEX = 3, slice thickness = 6 mm, spacing between slices = 1 mm) and sagittal contrast-enhanced T1-weighted spin-echo images (TR/TE: 3022/85 ms, FOV = 36 × 36 cm, NEX = 2 , slice thickness = 4 mm, spacing between slices = 1 mm), and Axial DWI (TR/TE: 4616/76 ms; FOV: 300 × 300 mm^2; matrix: 200 × 196; slice thickness/gap: 6/1 mm; b values of 0 and 800 s/mm^2) were obtained by using single-shot spin-echo EPI.

© 2020 Wu Q et al. *JAMA Network Open.*

eMethods 2. Mathematical description of the deep learning network.

The computational units in the deep learning network are defined as layers, which include convolution, activation, pooling and batch normalization. The details are explained as follows.

Convolution. Convolution is used to extract features from tumour images. Different convolutional filters can extract different features to characterize the tumor. Assuming matrix $I = \begin{pmatrix} I_{11} & I_{12} & I_{13} \\ I_{21} & I_{22} & I_{23} \\ I_{31} & I_{32} & I_{33} \end{pmatrix}$ is the mathematical representation of the tumor image, and matrix $K = \begin{pmatrix} k_{11} & k_{12} \\ k_{21} & k_{22} \end{pmatrix}$ is the convolutional filter. Then, the output of the convolution layer is $F = conv(I, K)$, where *conv* represents convolutional operation. This can be further understood as the following formula.

$$F = conv(I, K) = \begin{pmatrix} I_{11} * k_{11} + I_{12} * k_{12} + I_{21} * k_{21} + I_{22} * k_{22} & I_{12} * k_{11} + I_{13} * k_{12} + I_{22} * k_{21} + I_{23} * k_{22} \\ I_{21} * k_{11} + I_{22} * k_{12} + I_{31} * k_{21} + I_{32} * k_{22} & I_{22} * k_{11} + I_{23} * k_{12} + I_{32} * k_{21} + I_{33} * k_{22} \end{pmatrix}$$

The output F is called feature map. In this study, we used zero padding and convolutional stride of 1×1 to keep the image size after convolution.

Activation. After the operation of convolution, the result (feature map) will be activated by an activation function to obtain non-linear features; here we adopt the "ReLU" function[1] $ReLU(x) = max(0, x)$. When the input x is negative, the output of the activation function will be zero, and when the input is positive, the result will be equal to the input.

Pooling. To select representative features that are strongly associated with LNM status, non-relevant and redundant features need to be eliminated. This is achieved by pooling operation. Assuming the feature map is $F = \begin{pmatrix} 1 & 5 & 2 & 8 \\ 3 & 9 & 7 & 8 \\ 1 & 0 & 2 & 6 \\ 8 & 5 & 3 & 2 \end{pmatrix}$, whose size is 4×4, and pooling window is 2×2 with stride 2. The pooling operation will divide the matrix F into four disjoint small matrixes of size 2×2, and the maximum value of each small matrix will be extracted to form the result matrix $P = \begin{pmatrix} 9 & 8 \\ 8 & 6 \end{pmatrix}$.

Batch normalization. To accelerate the training process of the DL model, we use batch normalization[2] operation to normalize the feature maps from each convolutional layer. This strategy avoids gradient vanishing during training and therefore accelerates the learning process of the DL model.

Zero padding. Zero padding can add rows and columns of zeros at the top, bottom, left and right side of an image to feed into the DL model.

© 2020 Wu Q et al. *JAMA Network Open.*

eMethods 3. Development of the DL model and the hybrid model.

We developed an end-to-end DL model for LNM status prediction using MR images. Specifically, we designed a convolutional neural network as shown in **Supplementary Figure S2**. This DL model consisted of three parts (sub-network 1, 2, and 3 in **Supplementary Figure S2**). Sub-network 1 shared the same architecture with the first three building blocks in ResNet18.[3] The special structure (residual building block) in this network contributed to better performance than other plain deeper networks without increasing computational complexity.[4] Each residual block was the stack of multiple convolution layers, zero padding layers, and batch normalization layers with rectified linear activation function. In each residual block, a shortcut connection was used to combine the information between two distant convolution layers, which can optimize the learning process of the DL model by enhancing gradient flow in the network. Sub-network 2 was composed of six freshly added layers. Sub-network 3 was a fully connected output layer following the global average pooling layer in sub-network 2. When an MR image of the tumor was fed into the DL model, sub-network 3 can predict the LNM probability for the tumor. Since each tumor included multiple two-dimensional slices in MRI, we averaged the LNM probability of all image slices of the tumor to acquire the LNM probability for the patient. We defined the LNM-predicted probability from the DL model as the DL-score.

To enhance model training and improve the generalization ability of the DL model, we used transfer learning[5] to pretrain sub-network 1 by 14 million natural images from ImageNet dataset.[6] Afterward, we used image augmentation techniques, including random width and height shift, zooming, rotation, deformation, and flipping to enlarge our training dataset and to avoid overfitting. In previous reports, different MRI sequences showed different diagnostic performance in predicting LNM status.[7–10] Thereby, we compared the predictive performance of three MRI sequences to find the optimal sequence for LNM status prediction in CC. As a result, 5280 CET1WI, 1633 T2WI, and 1474 ADC map slices in the primary cohort were generated to fine-tune the DL model, respectively. Sub-network 2 and 3 were freshly trained using images from the primary cohort. All images (ROI_{tumor} and $ROI_{tumor+peri}$) were scaled to 64×64 voxel size and standardized by z-score normalization. To consider three-dimensional tumor information, we combined every three adjacent slices of MR images as input.

Since the DL model can mine high-dimensional information from MR images and the clinical model can reflect tumor information from clinicopathological aspects, we further developed a hybrid model to combine both two information to explore whether they can be complementary (sub-network 1, 2, and 4 in **eFigure 2**). This hybrid model concatenated the global average pooling layer of the previous DL model (sub-network 1 and 2) with the clinical variable (MR-LN status). Then multilayer perceptron with three hidden layers was added (size 6, 4 and 2) (sub-network 4). We defined the LNM-predicted probability from the hybrid model as the H-score.

© 2020 Wu Q et al. *JAMA Network Open.*

eMethods 4. Training process of the DL model and the hybrid model.

Model training aims at optimizing the parameters of the DL model and building the relationship between MR image and LNM status. The model training is an iterative process, which optimizes the model at each iteration until the model achieves the best predictive performance. At each iteration, we used cross-entropy as the cost function to measure the predictive performance of the DL model.

For the DL model, we froze the sub-network 1 first and trained the sub-network 2 by stochastic gradient descent (SGD) (learning rate = 0.00001). This is necessary because the sub-network 2 was initialized randomly and therefore generated large gradients, which may disturb the transferred layers in sub-network 1. After training the model on 20 epochs, we trained the full network in the DL model by root mean square propagation (learning rate = 0.00005) and the model converged after 30 epochs of training.

For the hybrid model, L2 regularisation was used to avoid overfitting during the training process. The weights of added layers in sub-network 4 were trained using resilient backpropagation with weight backtracking. All layers of the hybrid model were fine-tuned by SGD (learning rate = 0.001, decay = 0.9, and momentum = 0.9).

Our method was implemented in Python 3.6 and performed on a machine with an Intel Core i7-7700 CPU and 32 GB memory. The network training was implemented using Keras 2.2 with Tensorflow 1.7 backend and was accelerated on an NVIDIA TITAN XP GPU (12GB on-board memory). We used a batch size of 128 for model training, which meant 128 training samples were fed into the network at each iteration.

© 2020 Wu Q et al. *JAMA Network Open.*

eMethods 5. Details of the DL model visualization.

When the DL model is well trained, the network established thousands of inference paths that work together for the LNM status prediction. Given a tumor, we calculated the gradient of the predicted value with respect to the input image. This gradient told us how the predicted value changes with respect to a small change in tumor image voxels. Hence, visualizing these gradients helped us to find the attention of the DL model (defined as attention map in **eFigure 4**).

We used convolutional feature visualization technique[11] to acquire the feature patterns extracted by convolution layers. We defined these convolution features as DL-feature in **eFigure 5**. For each convolutional feature in the DL model, we input an image initialized with random white noise to observe the feature response. If the feature response reaches a maximum, the input image reveals the feature pattern extracted by the convolutional feature; otherwise, a back-propagation algorithm was involved to change the input image until the feature response reaches a maximum. Through this convolutional feature visualization method, we can understand the feature patterns extracted by each convolutional feature in the DL model.

We defined the maximum/minimum response convolutional feature of the last convolution layer as the positive/negative DL-feature in **eFigure 6**. In general, if a convolutional feature had different responses between node-negative and node-positive patients, it had the ability to discriminate the metastatic LN from non-metastatic LN.

© 2020 Wu Q et al. *JAMA Network Open.*

eFigure 1. Patient flowchart.

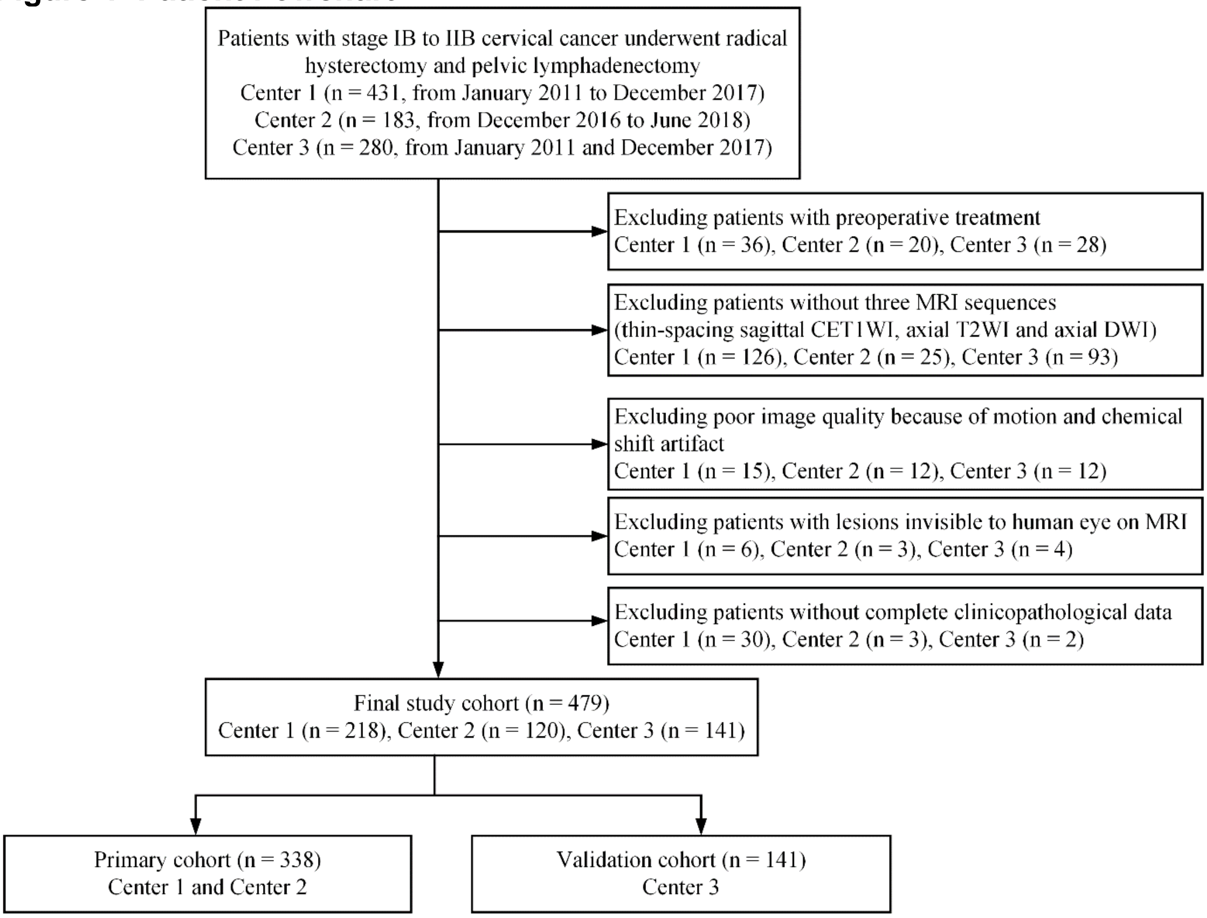

© 2020 Wu Q et al. *JAMA Network Open.*

eFigure 2. Architecture of the DL and hybrid model.

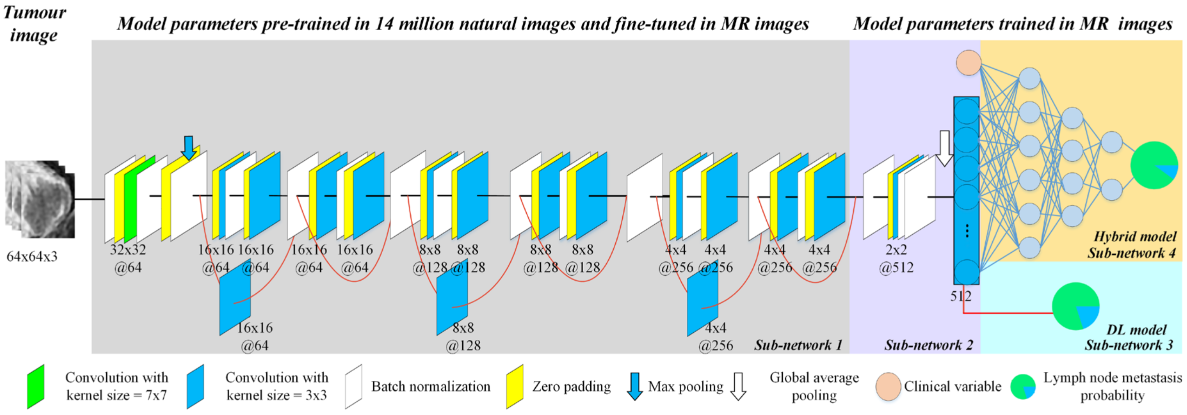

The two models share the same structure in the sub-network 1 and 2. The DL model consists of three parts (sub-network 1, 2, and 3). The principal architecture of the DL model is composed of convolution layers with kernel size 7×7 and 1×1, batch normalization, zero padding, and pooling layers. The output size after convolution layer is denoted as width × height @ filter (i.e., 32 × 32 @ 64 represents the output of the convolution layer is 32 × 32 × 4).

© 2020 Wu Q et al. *JAMA Network Open.*

eFigure 3. Performance of the DL score.

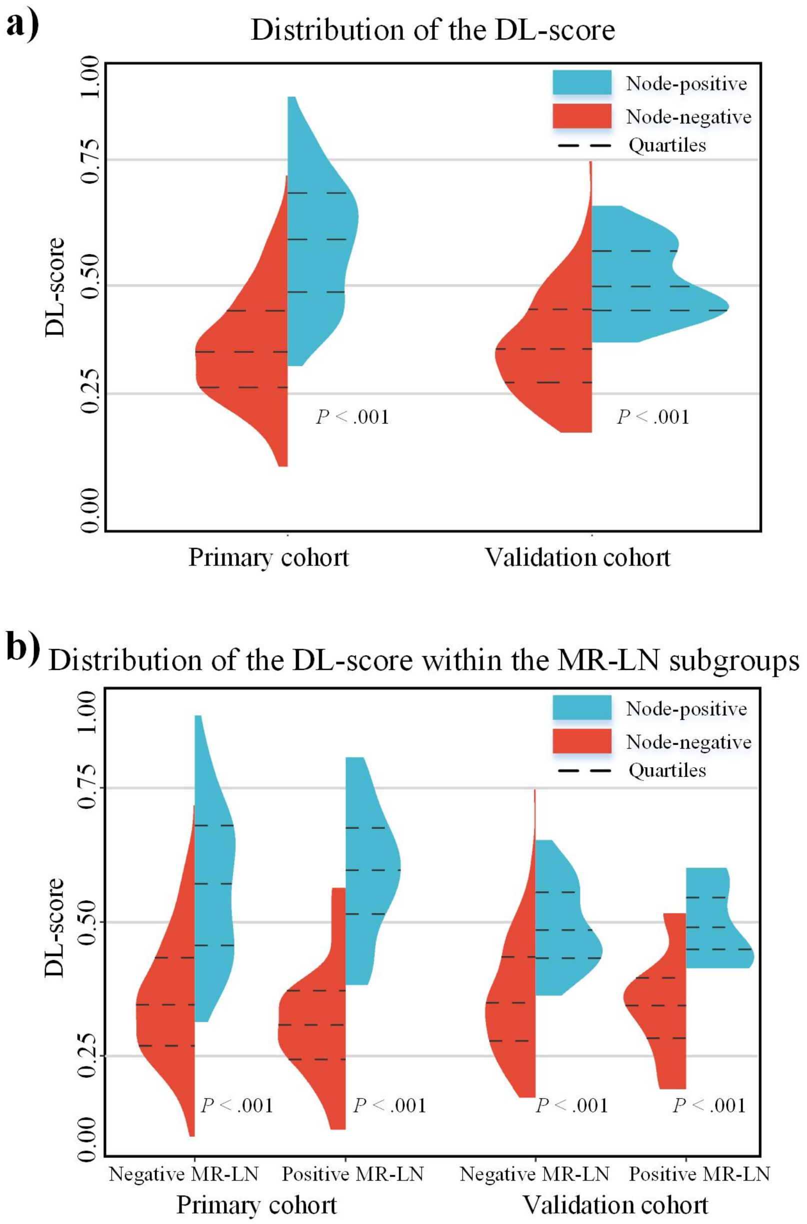

a) The DL-score from $CET1WI_{tumor+peri}$ between node-positive and node-negative patients in the primary and validation cohorts. DL-score in the primary cohort: 0.58 (IQR, 0.46-0.67) vs 0.34 (IQR, 0.27-0.43), *P* < .001; DL-score in the validation cohort: 0.47 (IQR, 0.43-0.56) vs 0.35 (0.27-0.43), *P* < .001.

b) The DL-score from $CET1WI_{tumor+peri}$ between node-positive and node-negative patients within the negative MR-LN and positive MR-LN subgroups in the primary and validation cohorts. DL-score among MR-LN-positive patients in the primary cohort: node-positive vs node-negative 0.60 (IQR, 0.52-0.67) vs 0.29 (IQR, 0.26-0.36), *P* < .001; DL-score among MR-LN-negative patients in the primary cohort: node-positive vs node-negative 0.56 (0.45-0.67) vs 0.35 (IQR,0.27-0.43), *P* < .001; DL-score among MR-LN-positive patients in the validation cohort: node-positive vs node-negative 0.45 (IQR, 0.43-0.56) vs 0.35 (0.29-0.38), *P* < .001; DL-score among MR-LN-negative patients in the validation cohort: node-positive vs node-negative 0.47 (IQR, 0.44-0.56) vs 0.35 (IQR, 0.27-0.43), *P* <.001.

© 2020 Wu Q et al. *JAMA Network Open.*

eFigure 4. Response area of representative patients.

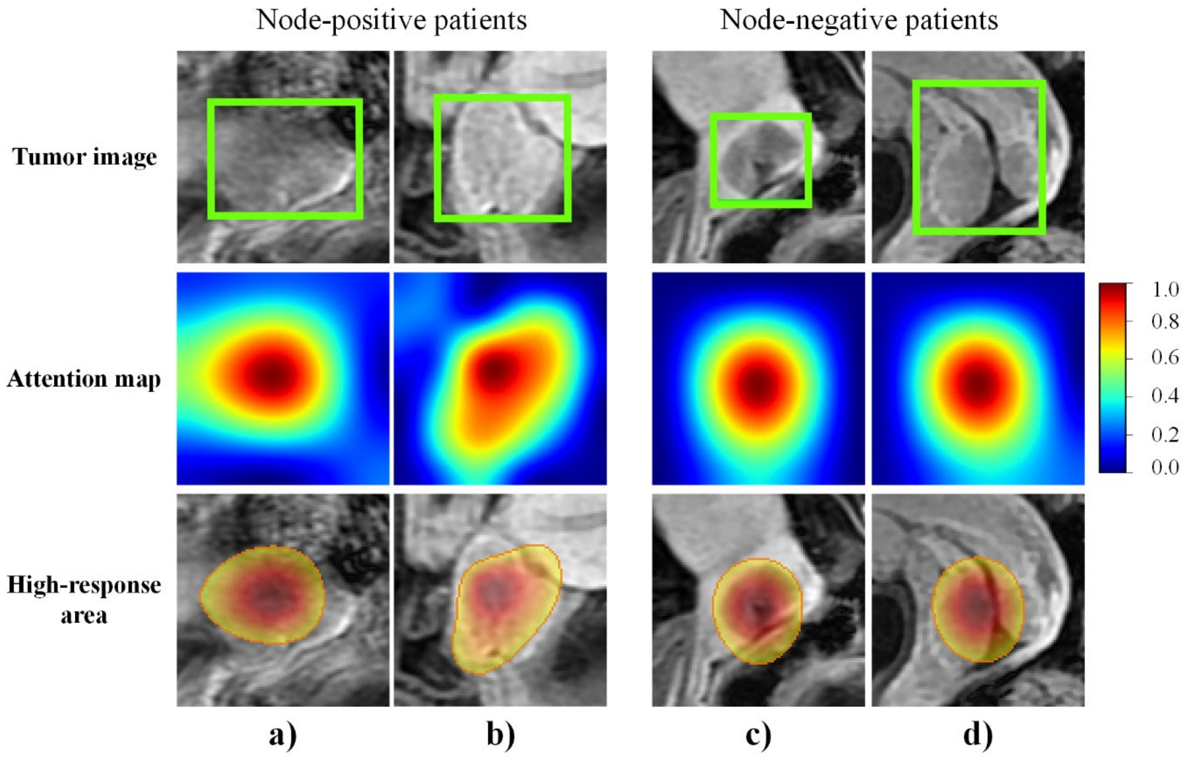

The first row shows sagittal CET1WI from two node-positive patients and two node-negative patients. The green box is $ROI_{tumor+peri}$. The second row shows the attention maps of the input tumor images. The high-response area in the third row is acquired by using 0.6 as the cut-off value.

© 2020 Wu Q et al. *JAMA Network Open.*

eFigure 5. The DL-feature visualization.

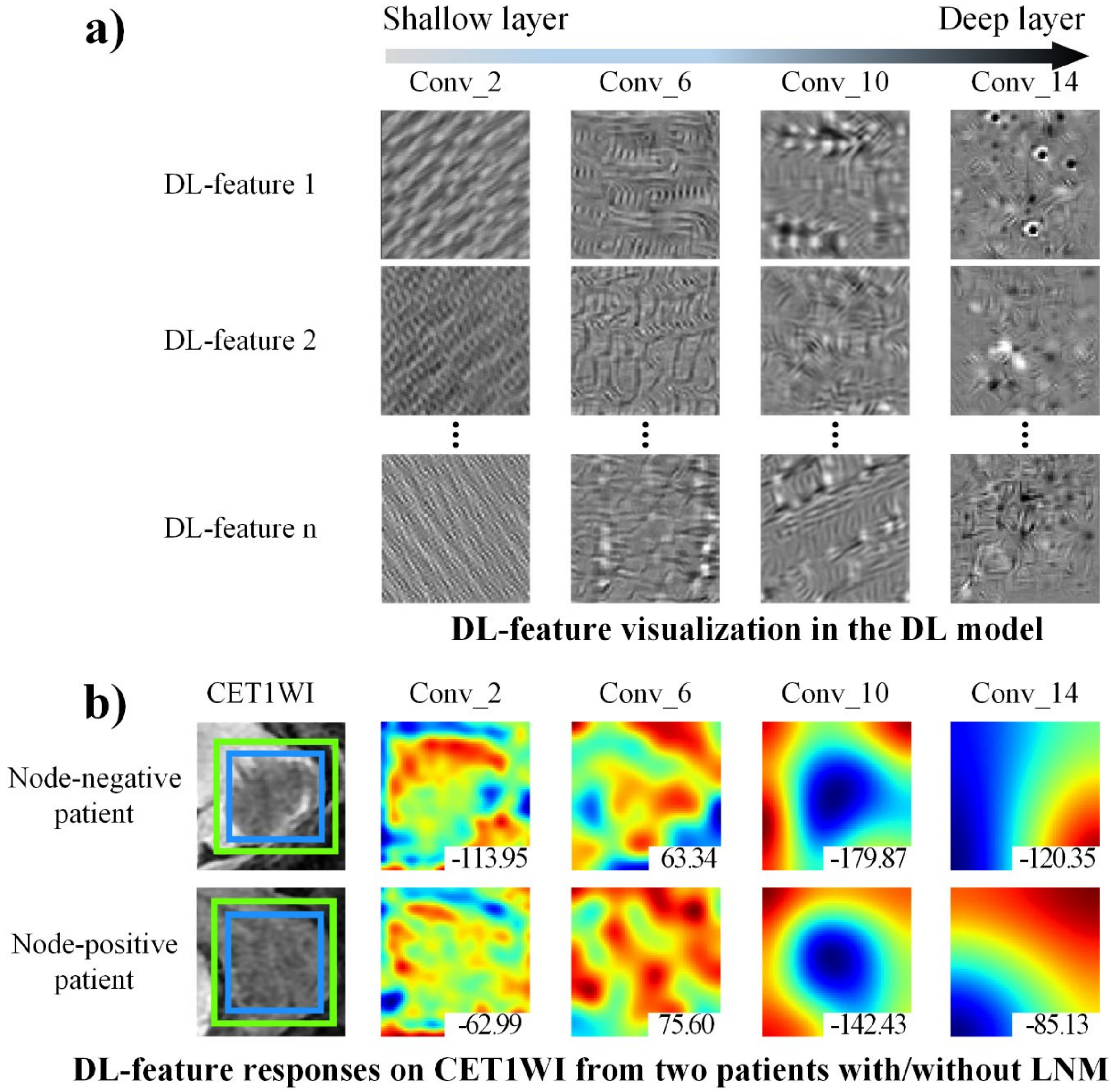

a) The DL-feature from the 2nd, 6th, 10th, and 14th (Conv_) layers of the DL model. Each convolutional layer has a different number of DL-feature, and we randomly select three DL-feature for visualization. b) The DL-feature responses on two representative patients from the validation cohort. The blue box in the first column is ROI_{tumor} and the green box is $ROI_{tumor+peri}$. Red and blue colors represent strong and weak responses, respectively. The number on the bottom right corner is the average value of the DL-feature response. When feeding two tumor images from two patients with/without LNM into the DL model, we can get different DL-feature responses.

© 2020 Wu Q et al. *JAMA Network Open.*

eFigure 6. The DL-feature analysis.

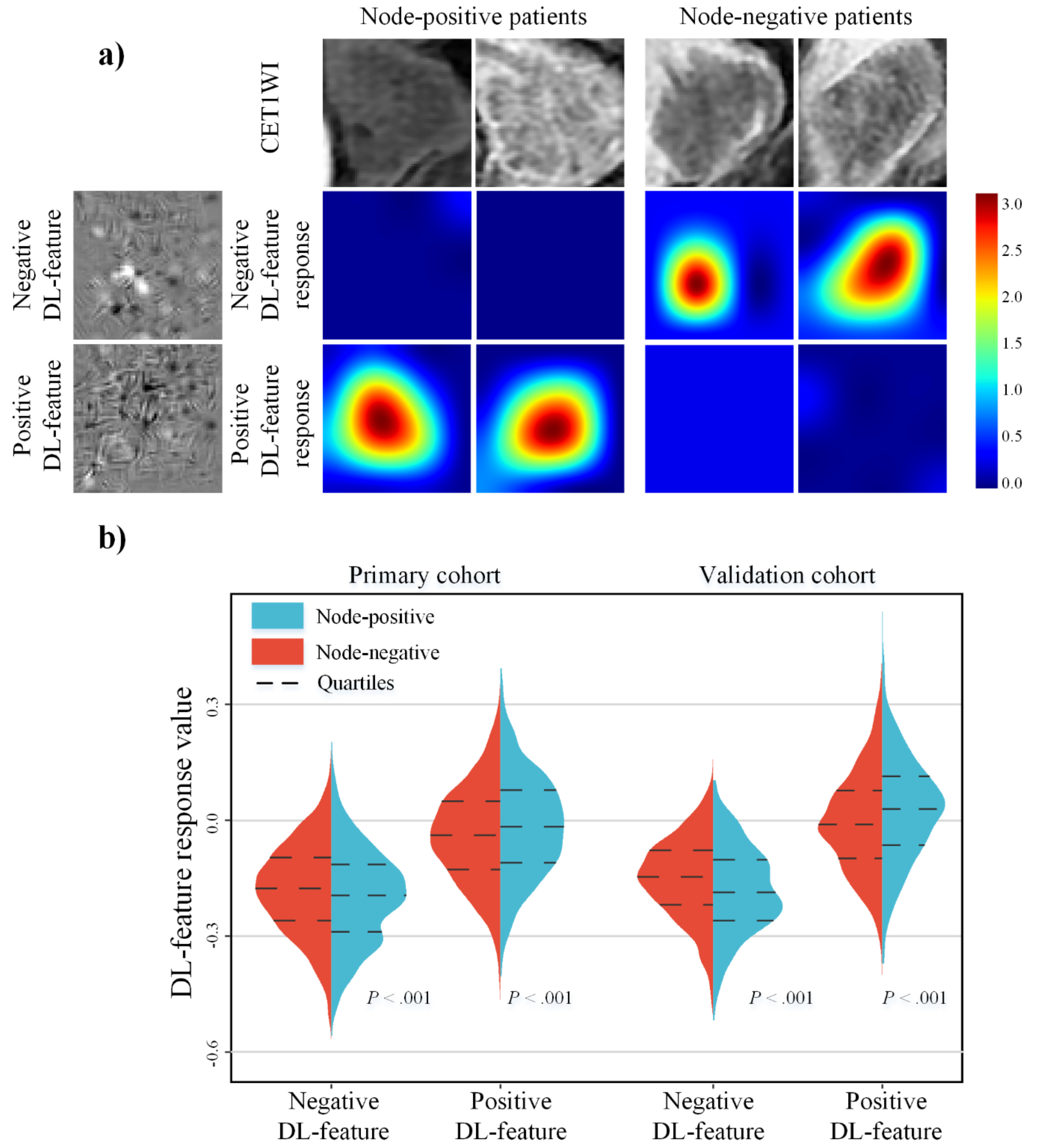

a) Response heat map of the negative DL-feature and the positive DL-feature in the four representative tumor images from sagittal CET1WI. All the images are from the validation cohort.

b) Response value of the positive DL-feature and the negative DL-feature in the primary and validation cohorts. DL-feature response among positive-DL-feature in the primary cohort: node-positive vs node-negative -0.014 (IQR, -0.104 to 0.077) vs -0.037 (IQR, -0.126 to 0.048), *P* < .001; DL-feature response among negative-DL-feature in the primary cohort: node-positive vs node-negative -0.195 (IQR, -0.291 to -0.114) vs -0.176 (IQR, -0.259 to -0.095), *P* < .001; DL-feature response among positive-DL-feature in the validation cohort: node-positive vs node-negative 0.030 (IQR, -0.059 to 0.111) vs -0.118 (IQR, -0.096 to 0.076), *P* < .001; DL-feature response among negative-DL-feature in the validation cohort: node-positive vs node-negative -0.182 (IQR, -0.257 to -0.103) vs -0.146 (IQR, -0.216 to -0.078), *P* < .001.

© 2020 Wu Q et al. *JAMA Network Open.*

eReferences

1. Krizhevsky, A., Sutskever, I. & Hinton, G. E. ImageNet Classification with Deep Convolutional Neural Networks. in *Proceedings of the 25th International Conference on Neural Information Processing Systems* 1097–1105 (2012).
2. Ioffe, S. & Szegedy, C. Batch Normalization: Accelerating Deep Network Training by Reducing Internal Covariate Shift. *arXiv e-prints* arXiv:1502.03167 (2015).
3. He, K., Zhang, X., Ren, S. & Sun, J. Deep Residual Learning for Image Recognition. in *2016 CVPR* 770–778 (2016).
4. Chang, K. *et al.* Residual Convolutional Neural Network for the Determination of IDH Status in Low- and High-Grade Gliomas from MR Imaging. *Clin. Cancer Res.* **24**, 1073–1081 (2018).
5. Yosinski, J., Clune, J., Bengio, Y. & Lipson, H. How transferable are features in deep neural networks? *arXiv e-prints* arXiv:1411.1792 (2014).
6. Deng, J. *et al.* ImageNet: A large-scale hierarchical image database. in *2017 CVPR* 248–255 (2017).
7. Wang, T. *et al.* Preoperative prediction of pelvic lymph nodes metastasis in early-stage cervical cancer using radiomics nomogram developed based on T2-weighted MRI and diffusion-weighted imaging. *Eur. J. Radiol.* **114**, 128–135 (2019).
8. Kan, Y. *et al.* Radiomic signature as a predictive factor for lymph node metastasis in early-stage cervical cancer. *J. Magn. Reson. Imaging* **49**, 304–310 (2019).
9. Yu, Y. Y. *et al.* Feasibility of an ADC-based radiomics model for predicting pelvic lymph node metastases in patients with stage IB-IIA cervical squamous cell carcinoma. *Br. J. Radiol.* **92**, 20180986 (2019).
10. Wu, Q. *et al.* Radiomics analysis of magnetic resonance imaging improves diagnostic performance of lymph node metastasis in patients with cervical cancer. *Radiother. Oncol.* **138**, 141–148 (2019).
11. Selvaraju, R. R. *et al.* Grad-CAM: Visual Explanations from Deep Networks via Gradient-Based Localization. in *2017 ICCV* 618–626 (2017).

© 2020 Wu Q et al. *JAMA Network Open.*
